# The private life of malaria parasites: Strategies for sexual reproduction

**DOI:** 10.1016/j.molbiopara.2021.111375

**Published:** 2021-07

**Authors:** Petra Schneider, Sarah E. Reece

**Affiliations:** Institute of Evolutionary Biology, Institute of Immunology and Infection Research, School of Biological Sciences, University of Edinburgh, Edinburgh, UK

**Keywords:** Gametocyte, Transmission, Fitness, Life history trait, Phenotypic plasticity, Adaptation

## Abstract

Malaria parasites exhibit a complex lifecycle, requiring extensive asexual replication in the liver and blood of the vertebrate host, and in the haemocoel of the insect vector. Yet, they must also undergo a single round of sexual reproduction, which occurs in the vector’s midgut upon uptake of a blood meal. Sexual reproduction is obligate for infection of the vector and thus, is essential for onwards transmission to new hosts. Sex in malaria parasites involves several bottlenecks in parasite number, making the stages involved attractive targets for blocking disease transmission. Malaria parasites have evolved a suite of adaptations (“strategies”) to maximise the success of sexual reproduction and transmission, which could undermine transmission-blocking interventions. Yet, understanding parasite strategies may also reveal novel opportunities for such interventions. Here, we outline how evolutionary and ecological theories, developed to explain reproductive strategies in multicellular taxa, can be applied to explain two reproductive strategies (conversion rate and sex ratio) expressed by malaria parasites within the vertebrate host.

## Highlights

• Sexual reproduction is obligate for malaria parasite transmission.
• Parasites have evolved “strategies” to maximise the success of sexual reproduction.
• Evolutionary and ecological theories can explain parasite reproductive strategies.
• Parasite strategies could undermine transmission-blocking interventions.
• Targeting parasite strategies offers novel opportunities for interventions.

## 1 Introduction

For all agents of infection (parasites, pathogens, microbes), denoted hereafter as parasites, transmission to new hosts equates to reproductive success, which is a major component of fitness. For malaria (*Plasmodium spp.*) parasites, reproductive success requires transmission from the vertebrate host to an insect vector that acts as the definitive host. A single round of sexual reproduction occurs in the vector’s midgut before development into sporozoites that can be transmitted onwards to new vertebrate hosts. Given that sexual reproduction offers an attractive target for disease control, the underlying mechanisms and how they have been shaped by natural selection remain remarkably enigmatic. This is, in part, due to the challenges of working with sexual stages and mosquito infections. However, progress is being made to uncover the genes and molecular processes underlying sexual stage commitment and development [[1], [2], [3], [4], [5], [6], [7], [8], [9], [10], [11], [12], [13]], and to understand how the reproductive strategies deployed by malaria parasites affect their fitness [[14], [15], [16], [17], [18], [19], [20], [21], [22]]. Explaining why parasites do things the way they do is important basic biology in its own right, but also key to forecasting how populations will respond to interventions. Here, we outline the application of evolutionary and ecological theories to explain investment into asexual stages, males, and females; how discoveries from malaria model systems translate to natural infections of humans; highlight areas that remain contentious; and suggest future directions. Whilst we focus on malaria parasites, the concepts we cover apply broadly to sexually reproducing parasite taxa.

**Fig. 1:**
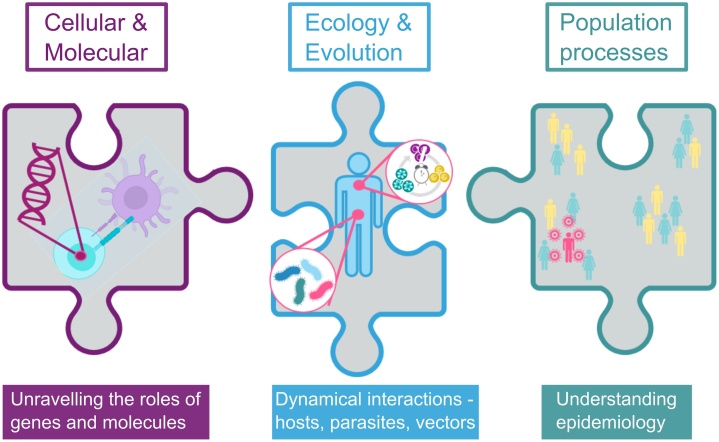
Interactions between hosts/vectors and parasites span all scales of biological organisation, from cellular and molecular processes to the physiologies, phenotypes and behaviours of individual hosts/vectors and parasite genotypes, to population-level processes. To fully understand infections it is necessary to integrate phenomena across these scales. Evolutionary ecology provides a useful point from which parasite phenotypes and their interactions with hosts/vectors can be discovered. Such discoveries demonstrate that there are causal molecular mechanisms underpinning strategies to be uncovered, which may reveal targets for interventions. Parasite phenotypes and their interactions with hosts/vectors also feedback with population-level processes, which together, explain and forecast epidemiological patterns.

**Fig. 2:**
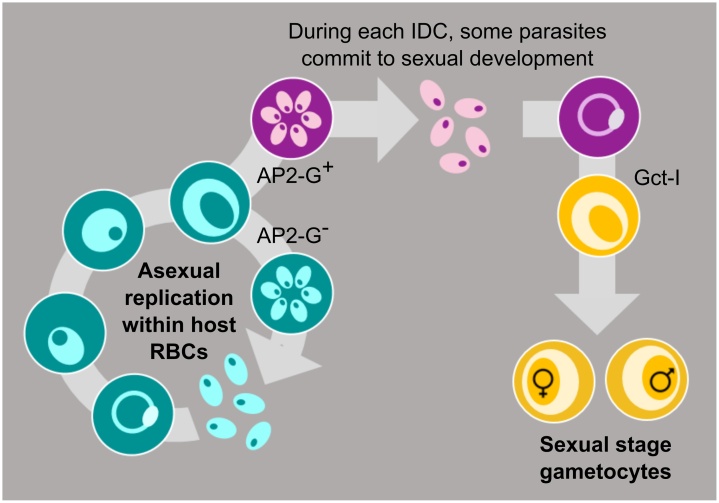
The intra-erythrocytic development cycle (IDC) of malaria parasites involves sequential rounds of asexual replication and the production of sexual stages (gametocytes) for transmission. Asexually replicating parasites (green) commit to sexual reproduction (purple, with AP2-G expression) generally in the cycle preceding conversion into gametocytes (yellow). Sexual commitment and gametocyte sex are not determined by the segregation of specific genes or sex chromosomes: a single haploid parasite within the vertebrate host can replicate to generate asexuals, male or female gametocytes. There are two resource allocation trade-offs involved: (i) investment into asexual stages versus gametocytes (conversion rate); and (ii) investment in male versus female gametocytes (sex allocation, measured as sex ratio).

For all parasites, within-host survival and between-host transmission are key components of fitness, so strategies to maximise these components are favoured by natural selection. Parasite fitness is a product of processes acting across both within- and between-host dynamics (Fig. 1). Asexual replication of malaria parasites within red blood cells (RBC, for a list of acronyms see Table 1) of vertebrate hosts (the “intraerythrocytic development cycle”, IDC) facilitates within-host survival and provides a source population to fuel the production of non-replicating sexual stages (“gametocytes”) for transmission (Fig. 2). The requirement of different stages for within-host survival and between-host transmission means that malaria parasites face life history trade-offs common to all sexually reproducing organisms: resources must be divided between growth/maintenance (i.e. asexual replication) and reproduction (i.e. production of gametocytes) [23,24]. In other words, a recently invaded blood stage malaria parasite can be either an asexual or a gametocyte, not both. Therefore, high investment in sexual stages early on in an infection might risk clearance due to insufficient investment in asexual replication in face of, for example, immune responses. As such, chronic infections with a low rate of transmission over a long duration could sum to greater “lifetime” fitness than high transmission during an infection of short duration [25].

**Table 1:** List of abbreviations.

AP2-G	Apetala 2-G
ApiAP2	Apicomplexan Apetala 2
APP	adaptive phenotypic plasticity
eIF2	eukaryotic initiation factor 2
FI	fertility insurance
GCPR	G-protein-coupled receptor
GDV1	gametocyte development 1 protein
GxE	genotype by environment interaction
HP1	heterochromatin protein 1
IDC	intraerythrocytic developmental cycle
LMC	local mate competition
NCC	next cycle conversion
PMT	phosphoethanolamine-N-methyltransferase
RBC	red blood cells
RR	reproductive restraint
SAH	S-adenosylhomocysteine
SAM	S-adenosylmethionine
SCC	same cycle conversion
TI	terminal investment

How malaria parasites resolve the “resource allocation” trade-off between investment into asexuals and gametocytes is called the “conversion rate” (Fig. 2). This is analogous to “reproductive effort” which quantifies how multicellular organisms divide their resources between their own growth/maintenance and investment in offspring [24]. Given the varied conditions malaria parasites experience during infections, evolutionary theory predicts that different conversion rates are required to maintain fitness in different circumstances [16,25]. Indeed, the ability of malaria parasites to alter conversion rate during infections, and in response to changes in conditions inside the host, appears to maximise fitness, i.e. be adaptive (Table 2; predictably plastic parasites) [17,21,26]. Investment into male versus female gametocytes (“sex allocation”) is another reproductive strategy that malaria parasites can alter (Fig. 2), and is also a resource-allocation trade-off because each gametocyte can either be male or female. Malaria parasites alter sex ratio during infections, and in response to changes in conditions inside the host, in manners predicted to maximise fitness [14,15,18]. Changes in host physiology could result in differential survival of certain parasite stages at certain points in infections and so, give the appearance of parasites actively altering sex allocation and conversion rate. This is likely to occur to some degree but there is ample evidence that variation in sex allocation and conversion rate can be well explained by parasites actively adjusting them rather than being a by-product of host factors (e.g. [14,17]). However, feedback between parasites and hosts will occur - how parasites adjust traits will affect how hosts respond to infection, which in turn alters the conditions parasites experience.

**Table 2:** Parasitologist’s guide to the ecology and evolution of life histories.

**From metazoans to malaria?**	Using theories developed to explain the strategies of multicellular organisms (such as plants, insects, vertebrates) to the behaviours of parasites that exist as many individual cells may intuitively seem inappropriate. However, each clone of genetically identical parasites within an infection is the evolutionary equivalent of an individual organism [113,114] because the fitness interests of close relatives are aligned. Thus, the transmission of a genotype early in an infection counts towards the fitness of their clonal progeny later in the infection, and vice-versa. In genetically diverse infections, parasites belonging to one genotype have no evolutionary interest in the fitness of unrelated genotypes and therefore each genotype is selected to maximise its own fitness, usually at the expense of competitors [115]. Thus, to understand parasite evolution, it is necessary to investigate the strategies of individual genotypes. This is particularly important if co-infecting genotypes are expected to behave differently to each other, for instance if they differ in competitive ability, or if strain-specific immune responses operate [116,117,118]. How a genotype orchestrates collective action is unknown but several pathways of parasite-parasite communication have been proposed [53,81,86,99,119] (Section 4).
**Predictably plastic parasites**	Adaptive phenotypic plasticity (APP) is the ability of a genotype to alter aspects of its phenotype and allows organisms to alter phenotype faster than evolutionary time scales would allow. Whilst the host provides parasites with an environment that is homeostatically controlled, the availability of resources (e.g. RBC), immune defences, and drug exposure can vary considerably throughout infections and between hosts, exposing parasites to a wide range of circumstances in a short period of time. Parasites that can plastically adjust traits, both according to the kind of host they find themselves in and as infections progress, have a fitness advantage over genotypes with inflexible (“fixed”) strategies [17,25,120] (Sections 2 and 3). However, plastic strategies come with the cost of having to maintain sensory mechanisms and mount responses, the risk of errors being made, and the possibility that the range of possible responses is more limited than if phenotypes are fixed [121]. Malaria parasite populations experience variation in multiplicity and host responses across transmission settings. Infections in low transmission settings may be less dynamic than in high transmission settings, resulting in a sufficiently stable within-host environment that parasites may longer need to pay the costs of plasticity and adopt fixed strategies. APP evolves when: environmental variation is frequently encountered and predictable; organisms can assess environmental change with reasonable accuracy; and the costs of environmental sensing are outweighed by the benefits of adjusting traits. If unpredictable environmental variation frequently occurs, a “bet-hedging” strategy may be better than APP [122,123]. Bet-hedging occurs when an individual produces diverse forms (usually offspring) that are suited to different types of environmental conditions. Only the forms that happen to be well matched to the conditions they encounter will thrive. Whereas the loss of unsuited forms decreases short-term fitness, the avoidance of extinction through ensuring some forms will maximise long-term fitness. Var gene switching in malaria is assumed to be a bet-hedging strategy [124]. However, bet-hedging is unlikely to apply to sex ratio or conversion rate because parasites adjust these traits in consistent and directional manners in response to the same change of circumstances (i.e. plasticity is repeatable rather than diversifying) [14,16,17].
**The footprints of hosts and parasites on phenotypes**	Parasite trait values reflect the combined impacts of parasite strategic decisions and the direct impact (by-product) of host conditions on the development and survival of asexuals, males, and females. For example, variation in sex ratio or conversion rate could result from the host causing selective death of certain stages and/or constraining the parasites’ ability to express an altered phenotype through resource limitation. Separating the relative contributions of host and parasite to infection dynamics matters because this reveals whose genes are under selection via the phenotypes they produce. For example, *P. chabaudi* produces consistent, genotype-specific patterns in sex ratio and conversion rate when measured in common garden conditions [14,17,78]. This suggests that genetic variation for these traits exists and is heritable (including via epigenetic modifications), which is a pre-requisite for parasite genes to be at least in part, in control of parasite phenotypes. To conclude that, e.g. parasites have increased conversion rate, it is necessary to exclude that survival of asexuals has decreased (and/or survival of gametocytes has increased) due to confounding changes in host immune responses [78]. Similarly, whether changes in the respective mortality rates of males and females masquerades as sex ratio adjustment must be discounted. Mathematical models can resolve these issues by estimating the stage- or sex-specific mortality rates required to fully explain observed changes in traits, and evaluating whether such rates are realistic ways for infections to operate. Experimental perturbations that induce a parasite response in the absence of environmental change (i.e. tricking parasites [52] or forcing phenotypes via conditional control of Ap2-G [71]) can also reveal the extent parasites control their phenotypes. Finally, the explanatory power of theories with a rich history in evolutionary ecology can also be leveraged: evaluating data against *a priori* predictions with a solid mathematical foundation is more compelling than generating *post-hoc* explanations for observations that invoke parasite adaptation.
**From models to humans?**	To test whether evolutionary theories for reproductive strategies apply to parasites, it is irrelevant whether parasites of humans or animals are investigated. However, to inform a medically, socially, and economically important disease such as malaria, it is necessary to verify whether evolutionary theories apply to human parasites. Details of parasite reproductive strategies are likely to vary across species [104], but if the same basic principles apply to lab models and diverse multicellular taxa, the assumption is that they also apply to human infecting *Plasmodium spp*. Simply observing that human parasite phenotypes correlate as predicted with variation in e.g. multiplicity of infection, is limited to revealing whether data are consistent with theory rather than testing its predictions. Elevating correlation to causation requires experimental perturbations of specific factors, demonstrating that parasites respond as expected, and that their responses return greater fitness than no response or alternative strategies. Testing how parasites respond to relevant environmental variation requires examining parasites in ecologically realistic settings. Clearly, this is more easily achieved using *in vivo* systems because the complexity of life inside a vertebrate host is captured. However, *in vitro* approaches do allow the environment to be tightly controlled to e.g. parse-out parasite and host contributions to parasite phenotypes and refine which cues parasites respond to. Natural infections of humans are defined by evolutionary and ecological realism but experimental possibilities are limited. Experiments are possible with natural infections of wild animals, including passerine birds [19,125] and lizards [43], and modest experiments might be compatible with human challenge models [126,127]. However, these systems are more challenging to work with than lab animal models, which therefore tend to be a more tractable “go-to” system for undertaking proof of principle studies.

Sex allocation and conversion rate are the focus of this article because, from an evolutionary perspective, they are the best understood reproductive strategies of malaria parasites (sections 2 and 3). This is largely due to the ease of translating the relevant evolutionary theories to the biology of malaria parasites (Table 2; from metazoans to malaria) and that sex allocation and conversion rate are relatively tractable traits to measure. There is uncertainty about the exact timing of conversion and sex allocation, and whether commitment to asexuals, males, and females all occur simultaneously. Even if resource allocation to asexuals versus males versus females occurs simultaneously, we treat sex allocation and conversion rate as two separate trade-offs. This is because, from the perspective of natural selection, the important trade-offs are the production of asexuals versus gametocytes and males versus females, not asexuals versus males or females.

Resource allocation decisions are one aspect of trade-offs that organisms are subjected to. Trade-offs more generally are likely to govern diverse processes underpinning transmission by malaria parasites. For example, the timing of the IDC may trade off maximising the maturity of gametocytes at the time-of-day mosquitoes bite against maximum asexual replication in the blood (e.g. [[27], [28], [29], [30]]). Of course, reproductive strategies are not just expressed in the vertebrate host – the production of gametes, mating, and zygote development all occur in the vector. Trade-offs may operate here too. Speedy male gametogenesis may be advantageous given that mating has to occur quickly, but might trade-off against the quantity of functional gametes [31,32]. And the duration of development in the vector (“sporogony”) may trade-off against the quantity or quality of sporozoites produced. Observations that avian malaria parasites become more transmissible upon exposure to mosquitoes [30,33] might also be explained by parasites fine-tuning their reproductive strategies. Investigating how male gametes locate female gametes in the blood meal, whether aggregation of gametocytes in the host is common and facilitates transmission, and whether females are equally likely to be fertilized by males of any genotype [31,[34], [35], [36]] could uncover new reproductive strategies as well as uncover new ways to target parasites and to make interventions robust to parasite evolution. However, beyond sex allocation and conversion rate, reproductive strategies remain a black box in need of investigation.

## 2 Sophisticated sex allocation

### 2.1 What is it?

Theory for sex allocation predicts the best ratio of sons to daughters for a parent to produce [22]. For malaria parasites, sex allocation is usually measured as the proportion of gametocytes produced during each IDC that are male (Fig. 2). Technically, this is the sex ratio, but as long as the same amount of resources are required to produce a male as a female gametocyte, then sex ratio is an accurate measure of sex allocation [37]. In many species, competitive or cooperative interactions between related individuals favour the evolution of unequal sex allocation, resulting in biased sex ratios [38]. For example, the consequences of competition between brothers is a common reason for sex ratios to be female biased and also translates to malaria parasites. Furthermore, observations that unmated female gametes adhere to each other *in vitro* [36], may indicate a cooperative interaction to facilitate their fertilisation, which would also favour a female bias.

### 2.2 What do parasites do and why?

**Fig. 3:**
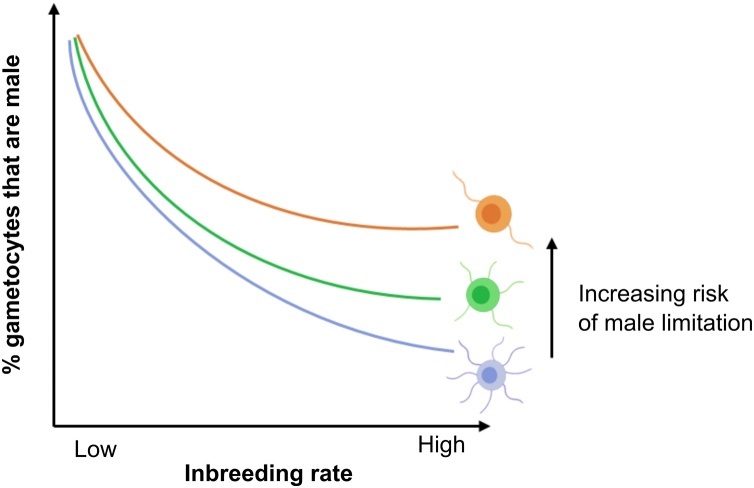
Sex allocation theory, applied to malaria parasites, predicts a negative correlation between the proportion of gametocytes that are male and the inbreeding rate. The number of co-infecting genotypes within a host determines the inbreeding rate their gametocytes will experience when taken up in a blood meal: inbreeding rate is low when gametes from mixed infections mate, whereas complete inbreeding occurs when all gametes belong to the same genotype. The precise relationship between inbreeding rate and sex ratio is affected by the degree of male limitation caused by, for example different fecundities of male gametocytes. For example, if males have maximum fecundity (8 gametes each, blue line) then extremely female biased sex ratios are predicted at high inbreeding rates, but if male fecundity is reduced (e.g. 5 gametes, green line; 2 gametes, orange line) then the extent of female bias becomes increasingly constrained at high inbreeding rates.

Gametocyte sex ratios are generally female biased, but the degree of bias varies during infections, between hosts, and across populations [37]. A key driver of sex ratio is the inbreeding rate, which is determined by the genetic diversity of infections [37,[39], [40], [41]] (Fig. 3; blue line). Complete inbreeding occurs when gametocytes from an infection composed of a single genotype are taken up by a vector. Within the blood meal, each female gametocyte transforms into a single female gamete and up to 8 male gametes are produced per male gametocyte. Fitness in single infections is maximised by female biased gametocyte sex ratios (up to 8 females per male) [37]. This reduces competition for mates between closely related male gametocytes, plus it provides the maximum number of females to mate with. In contrast, outcrossing occurs when gametocytes from mixed infections are taken up in a blood meal because gametes from multiple genotypes form a mating group. Males can fertilise unrelated as well as related females, and because each male gametocyte can mate with up to 8 females, increasing investment into males maximises a genotype’s genetic representation in the next generation.

The key concept underpinning gametocyte sex ratios is that in single genotype infections the optimal sex ratio maximises the *number* of parasite offspring, but when outbreeding, the fittest genotype is that with the highest *proportion* of matings. This theory, called “Local Mate Competition (LMC)”, is the best verified area of sex allocation theory [38]. Support for malaria parasites obeying the rules of LMC comes from experiments with several genotypes of the rodent malaria *P. chabaudi,* and the lizard malaria *P. mexicanum* [14,42,43]. During the acute phase of infections in naïve hosts, parasites plastically increase investment in males as more genotypes co-infect the host. Furthermore, *P. chabaudi* sex ratios vary according to the proportional representation of gametocytes belonging to each co-infecting genotype [14]. For example, the genotype contributing the most gametocytes to a mating group has the highest inbreeding rate and produces the most female biased sex ratio.

The explanatory power of LMC has limits because sex ratios vary during infections with a constant genetic diversity and some species (e.g. *P. mexicanum)* produce considerably higher sex ratios than LMC predicts [14,15,[42], [43], [44], [45], [46]]. This variation in sex ratio can be explained by “Fertility Insurance” (FI), an extension of LMC that accounts for the specific challenges faced by male gametocytes/gametes [37,47] (Fig. 3, orange and green lines). For example, when hosts are anaemic and/or gametocyte densities are low, extremely female biased sex ratios predicted at low inbreeding rates introduce the risk that insufficient males are taken up in the blood meal to fertilise the females present. Furthermore, transmission-blocking immune factors, and some drugs, have greater negative effects on the lifespan and fertility of males compared to females [[48], [49], [50]], which may also result in males being a limiting resource. Parasites are predicted to compensate for male limitation by producing a less female biased sex ratio than LMC alone predicts [37,47]. In keeping with these predictions, *P. vinckei* increases sex ratio in conditions mimicking host anaemia, and *P. mexicanum* is thought to suffer from male limitation because it generally produces fewer than 8 gametes per male gametocyte [32,44].

### 2.3 How do parasites assess circumstances?

**Fig. 4:**
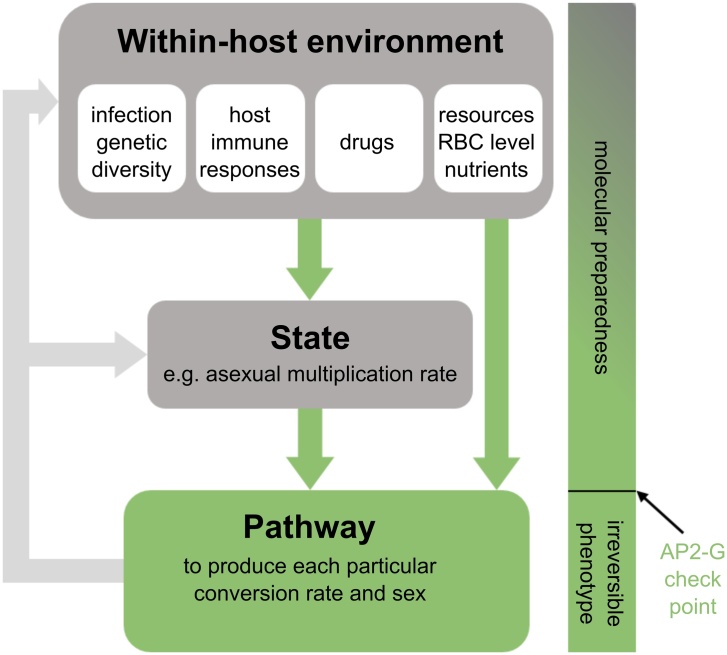
Schematic model for processes involved in sex allocation and conversion rate. Parasites adopt different trait values for sex ratio and conversion rate based on information about their circumstances (i.e. aspects of the within-host environment and their own state, green arrows). Pathways for sensing this information are unknown but could be part of “molecular preparedness” in which some parasite cells within each IDC appear primed for commitment / sexual differentiation. The AP2-G checkpoint likely occurs downstream of sensing pathways and reflects the parasite genotype actioning the sex ratio and conversion rate decision of each IDC. Infections are dynamic and it is important to note that the trait values for sex ratio and conversion rate produced at each IDC cohort will feed back into affecting conditions within the host and state for future IDC cohorts. For instance, adopting reproductive restraint (RR) improves state and results in greater exploitation of RBC.

That malaria parasites adjust sex ratio in response to the genetic diversity of infections, gametocyte density, and RBC availability is well-supported [14,15,51,52]. However, it is unknown how parasites transduce this environmental information into the molecular and cellular processes required to produce a particular sex ratio (Fig. 4). Signalling mechanisms like extracellular vesicles containing parasite genetic material [53] could be used to communicate information about identity and conditions within the host. However, evolutionary theory predicts that genetic diversity is better assessed via an indirect cue than directly differentiating between related and unrelated co-infecting parasite cells. This is because conflict between unrelated individuals favours “cheating strategies”, resulting in unreliable signals of identity [54,55]. Instead, sufficiently accurate environmental correlates (“proxies”) of the presence of co-infecting genotypes could hypothetically include strain specific immune responses or the detection of faster rates of resource depletion than expected for a focal genotype at low density due to competitive suppression. Similarly, it is unclear how parasites assess the presence of factors causing male limitation, but the development of immune responses against asexual stages may provide a useful proxy for the appearance of some transmission-blocking factors.

### 2.4 Does the theory apply to human malaria parasites?

*P. falciparum* does appear to follow the same rules of sex allocation as animal model species, but data are too scarce to compare to theory for other human parasites. Sex ratios of natural and experimental infections of *P. falciparum* are well-known for being female biased (reviewed in [56]). LMC is a likely explanation because sex ratio correlates with the average genetic diversity of infections across populations, and within-host sex ratios correlate with the genetic diversity of individual infections [[57], [58], [59], [60], [61]]. Given the general explanatory power of LMC for taxa as diverse as insects and plants, plus *P. chabaudi,* it would be surprising if *P. falciparum* was an exception to the rule (Table 2; from models to humans). Support for FI in *P. falciparum* comes from studies examining low gametocyte densities: these infections have the highest sex ratios [60,62,63], and transmission success is greatest at the highest sex ratios [64]. Furthermore, longitudinal-data indicate that FI is stronger in chronic *P. falciparum* infections [63], in keeping with observations in animal models [14,42,43]. In chronic infections, when parasites are at low densities overall, it may be harder for them to accurately assess the presence of others than detecting environmental cues for FI. *P. falciparum* sex ratios also correlate with the availability of RBC, as predicted by FI (e.g. [59,60]. Finally, the more rapid evolution of male-specific genes driven by host immune pressure, suggests that both plasticity and evolution help *P. falciparum* avoid male limitation [65].

## 3 Complex conversion rates

### 3.1 What is it?

Conversion rate is defined as the proportion of asexual stages within an IDC that become irreversibly committed and convert into gametocytes. This simple definition belies the difficulties in accurately measuring conversion [16,66]. Because gametocytes cannot be detected at the exact moment of commitment, gametocyte number must be linked back to asexual density in the IDC from which those gametocytes arose [16]. This is the case whether gametocytes are quantified by morphology or by any molecular marker for commitment or conversion that remains expressed for some time during commitment and gametocyte maturation. Thus, at least two appropriately temporally separated samples are required to estimate conversion rate. To avoid the difficulties in estimating conversion rates, other metrics including gametocyte prevalence (proportion of hosts carrying gametocytes), gametocytaemia (% of RBC containing gametocytes), gametocyte density (concentration), or the ratio of gametocytes to asexuals within a sample are often calculated [66]. These measures are useful estimates for transmission potential but do not convey information about conversion rates because asexual densities at the point of conversion are not accounted for. For example, low asexual densities with a high conversion rate could produce the same number of gametocytes as high asexual densities with a low conversion rate. The only exception occurs when comparing conversion rates across infections: if asexual densities at the point of commitment do not differ in these samples, then subsequent differences in gametocyte densities can reflect different conversion rates [66].

Estimating conversion from separate counts of gametocytes and asexuals is not perfect, and vulnerable to substantial bias, especially if parasites were in decline at the points of sampling, as is likely in drug treated or chronic infections [67]. Regular (i.e. at least daily) measurements improve conversion rate estimates because variable multiplication and mortality rates of asexuals, mortality of gametocytes, and RBC dynamics can be accounted for. Furthermore, the earlier that gametocytes/sexually committed parasites are detected [[68], [69], [70], [71]], the less opportunity gametocyte mortality has to bias conversion estimates. Finally, the recent discovery that parasites may not always commit to sexual conversion in the IDC before conversion takes place (next cycle conversion, NCC, Fig. 2), but can also commit and convert into gametocytes within a single IDC (same cycle conversion, SCC) [10], is yet to be incorporated into robust methods to estimate conversion. Simultaneous occurrence of NCC and SCC would make it difficult to determine from which asexual cohort gametocytes have arisen. However, NCC seems to be the more common pathway, with SCC occurring rarely *in vitro* and its existence *in vivo* remains to be verified.

### 3.2 What do parasites do and why?

**Fig. 5:**
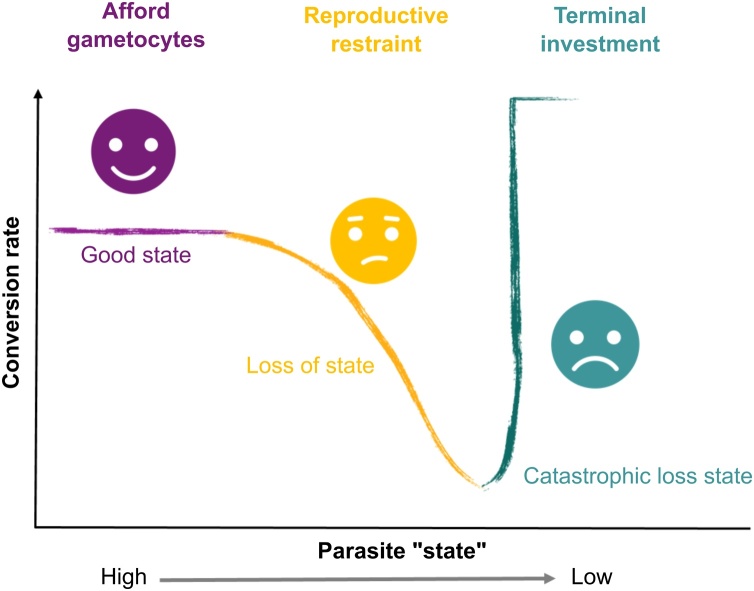
Patterns of conversion rate follow a continuous, non-linear “reaction norm” in response to how parasites’ circumstances change during infections. Parasites in good state (e.g. replicating exponentially) can afford to invest in gametocytes, whilst a moderate loss of state requires parasites to reduce conversion (reproductive restraint, RR) to facilitate within-host survival and future transmission. During infections, parasites adopt variable but generally low conversion rates as state repeatedly improves and declines in line with dynamic features of infections, including immune responses, drug treatment, and changes in the age structure and density of RBC. However, in response to a catastrophic loss of state that likely eliminates the infection or imminent host death, parasites should maximise conversion (terminal investment, TI) because prioritising short-term transmission provides the best chance of fitness returns.

Across Plasmodium spp., conversion rate is generally low but also varies during infections [21,72,73]. Explaining patterns of conversion is a long-standing puzzle that is important to solve because the density of gametocytes represents transmission potential, and conversion also affects the production of symptom-causing asexual stages. We proposed that plastic conversion rates allow parasites to cope with the changeable circumstances they experience during infections [16,74]. We predicted that conversion, like reproductive effort of multicellular organisms, should follow a pattern (“reaction norm”) that is non-linear with respect to the concept of parasite “state” (Fig. 5) [16,17,75,76]. In evolutionary ecology, the term “state” captures the physiological condition and survival prospects of an organism. For malaria parasites, state may be best interpreted as the multiplication rate of asexual stages belonging to a parasite genotype within an infection (Table 2; from metazoans to malaria). For example, state is high when multiplication rates are high (for example in naïve hosts before significant immune defences and RBC limitation occur), and state is reduced – often catastrophically – by drug treatment.

When parasites experience a mild-moderate loss of state they should reduce conversion ("reproductive restraint”, RR) to prioritise asexual multiplication, which promotes within-host survival. That malaria parasites adopt RR in response to stressors such as low drug doses and within-host competition experienced in mixed infections has been experimentally verified [17,77,78]. Thus, RR is another strategy (along with antigenic switching and dormancy, a phenomenon where parasites temporarily pause development during the IDC) to invest in a longer duration of infection and garner the fitness returns of future transmission opportunities [16,17]. However, when parasites experience a loss of state so catastrophic that RR is unable to ensure within-host survival, the best strategy is to maximise short-term transmission by increasing conversion (“terminal investment”, TI). For example, when clearance is likely due to immune responses, drugs, or impending host death, TI has the potential to return some fitness but RR does not [16,17,76,79]. TI occurs in response to high drug doses, immune responses and spent medium (reviewed in [17]). Whilst adverse circumstances reduce parasite fitness overall, parasites adopting the best conversion strategy (whether that is RR or TI) have higher fitness relative to those that do not adjust conversion. It is important to note that state is likely to cycle between high and low throughout the majority of an infection because parasites wax and wane along with fluctuations in for example, RBC availability and immune responses, leading to repeated bouts of RR and higher conversion (Fig. 5).

TI has long been assummed to explain plasticity in conversion rates; elevated conversion is typically interpreted as parasites exhbiting a response to stress, but this view is incomplete [17]. Many studies reporting a drug-induced increase in conversion (often based on alternative metrics such as % gametocytes) may miss RR because low doses were not used. Additionally, RR can only be observed if conversion rates have scope to be reduced, i.e. when parasites are in good enough state that a loss of state is not catastrophic (Fig. 5). Finally, observations that conversion rates increase in hosts recovering from anaemia, or when cultures are supplemented with reticulocytes, are better explained by an influx of resources improving parasite state and releasing them from RR, as shown for *P. chabaudi* [26]. Overall, the predicted relationship between conversion rate and state is best-supported by drug-dose response experiments using several genotypes of *P. chabaudi* that incur a loss of state, over a range of 0 to >99 % of asexuals killed [17].

Most evidence for plasticity in conversion rate comes from drug challenges (reviewed in [17]), but drugs do not feature in the evolutionary history of many of the strains used in these studies. Indeed, parasites do not directly respond to the presence of drugs, but to the extent that drugs reduce parasite number [77], adding weight to the notion that parasites have evolved to respond to changes in state. Similarly, kin discrimiation has been observed in malaria parasites [14] and informs conversion strategies, most likely by how the degree of competition generated by co-infecting genotypes alters parasite state [17]. In addition to monitoring changes in state, *P. berghei* and (multiple genotypes of) *P. chabaudi* change conversion in response to whether RBC density is increasing or decreasing [17,26,44]. In general, parasites in good state adopt higher conversion when RBC are increasing and are more likely to respond to a loss of state with RR when RBC density is increasing [17]. Such a scenario may apply to the post-peak phase of infections when hosts are recovering from anaemia. Thus, as for sex ratio, plasticity in conversion rates correlates with variation in state and levels of RBC resources.

**Fig. 6:**
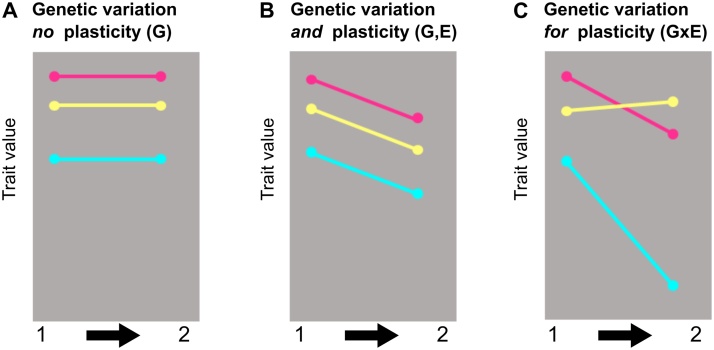
Both plasticity and genetic variation influence how phenotypes are affected by a change in conditions. A-C illustrate different ways (“reaction norms”) that three (pink, yellow, cyan) hypothetical parasite genotypes alter a trait (phenotype) in response to a shift in their circumstances (e.g. environmental variation). Genotypes differ in the trait values they exhibit but do not alter their trait in response to a change in circumstances in (A), whereas this trait is sensitive to environmental change in (B, C). Each genotype alters its phenotype by the same extent in response to environmental change in (B), but their responses differ in (C, lines are not parallel).

Testing how a change in state affects conversion rate is best assessed from relative changes in conversion, either within an infection/culture or through perturbatations of state for replicate infections/cultures. Data should be interpreted with reference to the following. First, any baseline (constitutive) level of conversion may constrain the degree of RR parasites can adopt. This baseline level may vary between genotypes (Fig. 6). Second, whether parasites respond to a given perturbation with RR or TI depends on the switch point between these two strategies. Genetic variation for the switch points is likely to occur (Fig. 6) and for *P. chabaudi,* this varies from 43 to 85% loss of asexual stages depending on the genotype [17]. Third, given that TI is a “last ditch resort” for parasites in dire circumstances, the level of conversion achieved may not exceed levels observed when in good state [76]. This is because the maximum possible conversion will be affected by resource limitation and how fast the number of asexuals available to contribute to conversion declines relative to sampling. Furthermore, only a subpopulation of asexual parasites in each IDC may be capable of conversion. Such a pre-set cap might be advantageous because it prevents parasites from accidentally converting so much they prematurely end their infection.

### 3.3 How do parasites assess circumstances?

As a general rule, parasites should adopt the same response to a change in state, regardless of what causes state to change (e.g. immune responses, within-host competition, drug treatment, resource limitation). Whether parasites infer state from their density or multiplication rate is unknown. However, density alone is unlikely to be informative: parasites could find themselves at a low density for reasons that require different conversion rates - at the start of infections when RR is needed to help parasites establish (and improve state), or due to catastrophic circumstances that demand TI (because there is little hope for state to improve). Cues for state that parasites could detect include changes in circulating levels of parasite derived products, host immune responses to infection, and/or micronutrients (e.g. [52]). Potential candidates include (or could be signalled by) soluble factors, extracellular vesicles, Ca^2+^ signalling pathways and GCPR-like proteins, phophorylation of eIF2 and kinases [9,53,[80], [81], [82], [83], [84], [85], [86], [87]].

### 3.4 Does the theory apply to human malaria parasites?

*P. falciparum* does appear to follow the same state-dependent conversion rules as animal model species, but data are too scarce to compare to theory for other human parasites (Table 2; from models to humans). *P. falciparum* gametocytes have an unusually long maturation duration and lifespan, leading to the suggestion that their conversion strategies should be different to other *Plasmodium* spp. (e.g. [88]). However, organisms as diverse as beetles and birds which have vastly different lifespans adopt either RR or TI according to their prospects for future reproduction [[89], [90], [91]]. In addition, the IDC of *P. falciparum* is ∼48 h, so although RR will alter an infection’s fate slower than for parasites with a shorter IDC duration, *P. falciparum’s* IDC is faster than the maturation period for gametocytes allowing it to alter its fate faster by RR than TI. This suggests that *P. falciparum* must prepare well in advance to benefit from adopting TI and therefore may have a more conservative switch point between RR and TI than rodent models. *P. falciparum, in vitro* and *in vivo,* responds to perturbations of immunity, drug treatment, competition, and RBC, in manners consistent with RR and TI, even if not interpreted as such by the authors (e.g. [92], reviewed in [17]). Furthermore, conversion by *P. falciparum* is modulated by factors relating to state including density-dependent sensitivity to parasite-derived products, extracellular vesicles, spent medium, depletion of nutrients or RBCs, and the impact of drugs [12,52,77,87,[93], [94], [95], [96], [97], [98], [99]].

## 4 Part-solved mysteries and future directions

### 4.1 Mechanisms underpinning plasticity in conversion rate and sex allocation

How do parasites detect their circumstances, determine what sex ratio or conversion rate (“trait value”) to produce, and then action the production of specific trait values? Rapid progress is uncovering the molecular pathways involved in the latter, but how these mechanisms integrate with the upstream processes of environmental sensing and decision making remain mysterious (Fig. 4). For example, what information do parasites detect to monitor changes in state, RBC density, and genetic relatedness of co-infecting parasites / multiplicity of infection, and do they use different parameters to inform sex ratio and conversion rate decisions? If parasites are unable to directly “count” their number in each IDC, or RBCs etc., they are limited to using cues that correlate with changes in state and their within-host conditions. Basing decisions on indirect proxies introduces the risk of making the wrong decisions but can be advantageous by providing advance notice that environmental change is imminent. Further, how is information about the environment and state transmitted throughout each IDC cohort belonging to the same genotype (Table 2; from metazoans to malaria)? Perhaps cell-cell communication [53,81,86,93,94] disseminates instructions throughout each cohort, though this is vulnerable to eaves dropping and exploitation by co-infecting genotypes [54,55]? Or perhaps asexual cells sense and respond to environmental/state cues as individuals but in a probabilistic manner, such that on average across the cohort, the best trait values for sex ratio and conversion rates manifest. Both of these scenarios are compatible with recent suggestions that only a sub-population of asexuals within each IDC have the potential to leave the asexual cycle [1,2,8,10,71,100,101]. Such a strategy may be a safety catch, preventing all asexuals converting and/or all gametocytes differentiating into the same sex. Whatever the strategy, the population of asexuals within an IDC cohort must retain flexibility in the proportion of parasites that become gametocytes of each sex, whilst the pathways that individual parasite cells follow are defined by developmental switches, making their fate irreversible (Fig. 4).

Identifying cues that stimulate parasites to adjust sex ratio and conversion may be facilitated by uncovering the molecular pathways involved (Fig. 4). Sensing messengers may include Ca^2+^ activated proteases, cyclic nucleotide-dependent kinases, Ca^2+^-dependent kinases and metabolic enzyme effectors, GPCR-like proteins and small GTPases [9,53,[80], [81], [82], [83], [84], [85], [86]]. Equally, insight into cues could open the black boxes of how the parasite senses environmental change as well as the molecular mechanisms involved in sexual development [56]. In the last decade, pathways involved in sexual commitment and conversion have been partially elucidated [[3], [4], [5],^7^,[10], [11], [12],^70^]. Whilst progression through various developmental stages seems largely under control of members of the ApiAP2 transcription factor family [9,102,103], disruption of ApiAP2 members does not appear to affect sex ratios [103]. The involvement of AP2-G as a master regulator of commitment and the regulation of AP2-G expression through silencing by H3K3me3 and HP1, occurs across species [3,4,[6], [7], [8], [9],^11^,^103^]. However, multiple studies reveal gametocyte-specific genes are expressed before AP2-G, suggesting that there is a pre-commitment program that may prepare (potentially a subset of) asexuals for future commitment [1,2,8,10,71,100,101]. Without AP2-G, gametocyte transcripts may not be stabilised and commitment aborted [1,101]. Thus, AP2-G expression within an IDC provides a readout for conversion when related to the number of asexuals in that cohort, but may not be instrumental in determining what the conversion rate should be. Furthermore, the timing of sex allocation is unclear, and it may occur prior to AP2-G mediated processes, and thus may also be part of a program of molecular preparedness (pre-commitment) that takes place before the irreversible AP2-G checkpoint [1,2,8,10,71,100,101] (Fig. 4). How plasticity in sex ratio and conversion are assured is unknown, but the potential for multiple ApiAP2 family transcription factors, and undiscovered transcription factors, to bind to the same motifs could generate diversity in transcriptional patterns.

Identifying the molecular players is further complicated by the potential for species differences. For example, some processes upstream of AP2-G (e.g. GDV1 and its antisense RNA regulation of AP2-G expression [5,104]) vary between species and some genes that are important for commitment in *P. falciparum* are absent from rodent malaria genomes [5,12,88,104,105]. This includes a putative link between environmental sensing (response to lysoPC) and orchestrating a conversion rate decision (AP2-G expression) through the phosphoethanolamine-N-methyltransferase (PMT) pathway. Limitation of phosphocholine precursors (e.g. lysoPC) activates the PMT pathway for phosphocholine synthesis. This pathway uses the methyl donor S-adenosylmethionine (SAM), which may decrease the amount of SAM available to repress AP2-G, thus allowing commitment to gametocytes [106]. The process also generates S-adenosylhomocysteine (SAH) which inhibits methyltransferases and could interfere with heterochromatin maintenance including HP1-driven control of the AP2-G expression (reviewed in [107]). Whereas PMT is absent in rodent malarias, these species are still responsive to SAH, so rodent parasites and *P. falciparum* likely use (partially) different pathways to action the same strategies. This is not uncommon: multicellular species use widely varying mechanisms for environmental sensing and altering reproductive strategies [22]. Future research could capitalise on species differences within the *Plasmodium* genus [104] to elucidate whether the details of mechanism are directly related to variation in gametocyte developmental programs and/or differences in strategies (e.g. the switch point between RR and TI).

### 4.2 Adaptive value

Sex allocation has been referred to as a “jewel in the crown of evolutionary biology” due its compelling predictive power [108], but significant unknowns remain. First, are the fitness benefits of adjusting sex ratio as predicted by theory borne out in reality? For instance, do female biased sex ratios return higher fitness for parasites in single than mixed infections, and does FI ameliorate the fitness costs of male limitation? Similar questions can be levied at conversion rates – does RR improve prospects of within-host survival at a cost to short term transmission, and vice-versa for TI? Across evolutionary ecology as a whole, quantifying fitness consequences is notoriously difficult. We note this deficit applies broadly in parasitology too – many parasites traits that are implicitly assumed to be adaptive (such as translational repression, the rate of var gene switching) also lack hard evidence of fitness consequences (Table 2; the footprints of hosts and parasites on phenotypes).

Assessing fitness consequences requires testing whether the fitness returns of the predicted sex ratio / conversion rate strategy for a given circumstance are higher than biologically plausible alternative trait values. This is complicated because a fair test requires comparing the consequences of all the trait values under the same conditions. Such tests can establish that variation in trait values are adaptive, and not solely a result of host factors giving the appearance of parasites actively altering phenotypes (Table 2; the footprints of hosts and parasites on phenotypes). But how can parasites be induced to produce a range of different conversion rates (or sex ratios) when their circumstances are held constant? Assuming parasites use proxies to interpret their circumstances, once cues are identified, these can be provided in different concentrations to trick parasites into adopting a range of sex ratios (or conversion rates) regardless of their actual circumstances. Decoupling a real change in circumstances from stimulation to change sex ratio (or conversion rate), enables testing whether the predicted strategies return the highest fitness under controlled conditions (called a “common garden”). For example, using standardised mating culture conditions, the mating success of *P. berghei* across the full range of gametocyte sex ratios (0–100 % males) does reveal that a female bias returns the highest reproductive success [14], and a male bias yields most offspring when *P. falciparum* experiences FI [64]. The conversion rates of *P. chabaudi* observed across a gradient for state coupled with the consequences for the resulting dynamics of asexuals and gametocytes suggests that RR and TI affect short term survival within the host and between-host transmission potential as predicted [17]. Another approach would be to directly interfere with the molecular mechanisms involved in environmental sensing and/or sex determination (for example, forcing / repressing AP2-G expression (e.g. [71]) to coerce parasites into adopting varied sex ratios or conversion rates in a common garden.

## 5 Evolutionary considerations for interventions

Sexual reproduction of malaria parasites involves huge bottlenecks in terms of parasite densities (from asexuals to gametocytes, and to subsequent ookinetes and oocysts within the mosquito vector), making the processes involved attractive targets for interventions. However, it is important to consider how the sophistication with which malaria parasites appear to optimise conversion and sex ratio could erode such interventions [109,110]. For example, in response to drug treatment, RR makes infections harder to clear but TI enhances short-term transmission. This applies both to short-term (i.e. plastic) responses and longer-term (i.e. evolutionary) responses (Table 2; predictably plastic parasites). Predicting – and preventing – clinically unfavourable responses to interventions such as drugs, vaccines, and vector control is desired and requires knowledge about heritable genetic variation, pleiotropy, and plasticity in parasite phenotypes (Fig. 1).

### 5.1 Genetic variation

Heritable genetic variation is the raw material that natural selection acts upon. Genetic variation exists for both sex ratio and conversion rate, across different genotypes, both for model systems and *P. falciparum* (Fig. 6). Moreover, genotypes differ in how much they alter sex ratio and conversion rate in response to a change in circumstances (genotype by environment interactions, GxE, Fig. 6C) [14,78]. For example, different *P. chabaudi* genotypes vary in their switch point from RR to TI when state deteriorates [17]. GxE interactions are thought to maintain genetic variation in the face of natural selection and can also expose genetic variation to natural selection when the environment changes [111]. The patterns for GxE illustrated in Fig. 6C show how a hypothetical population of three genotypes (yellow, cyan and pink) alter a trait value (for example sex ratio) in two different conditions (for example pre- and post-vaccination of all hosts with a transmission-blocking vaccine). If higher sex ratios return greater fitness to parasites, the vaccine reduces the fitness of the cyan and pink genotypes (because for example, their ability to respond effectively to FI has decreased), without much effect on the yellow genotype. Because the genotypes’ trait values (sex ratio), and thus their fitness differences, are more widely spread in vaccinated hosts, selection can change gene frequencies of the parasite population faster than pre-vaccination (i.e. the loss of the blue genotype will be faster). Thus, depending on the frequencies of the genotypes before vaccination (i.e. if blue is common), the vaccination program could lead to a short-term reduction in transmission but this gain for public health could soon be lost as the fittest genotype rises in frequency (i.e. yellow).

### 5.2 Pleiotropy

Pleiotropy refers to the phenomenon of a gene influencing multiple traits. Thus, selection to alter reproductive strategies could result in correlated traits being altered too, and vice versa. For example, more virulent genotypes might require a greater loss of state to switch from RR to TI because they are better at recovering lost state, and so, they can withstand greater loss in number before TI is necessary. If conversion rate and virulence are pleiotropically linked then selection pressures such as drugs and multiplicity of infection that select for higher virulence will also affect conversion rate. In addition, conversion rate and var gene switching may be linked through shared mechanisms like HP1 involvement in the epigenetic modification of H3K9me3 in blood and mosquito stages [1,11,112]. If so, selection for plastic conversion rates may also affect the rate of var switching (and vice versa), with unforeseen consequences for immune evasion, virulence and chronicity. These hypothetical scenarios illustrate the need to know whether other medically relevant traits correlate with sex ratio and conversion rate strategies.

### 5.3 Plasticity

In theory, plastic strategies (Table 2; predictably plastic strategies) can constrain or facilitate evolutionary change in populations. For example, plasticity in conversion rates may affect the emergence and spread of “classical” drug resistance in malaria parasites [20]. By adopting RR (in response to low drug doses) parasites can partially compensate for drug-induced fitness loss. On one hand, an adaptive phenotypic plasticity (APP) response reduces the strength of selection for resistance imposed by drugs, compared to selection on non-plastic parasites who suffer greater fitness losses. On the other hand, RR maximises asexual density, which maximise the number of genomes in which *de novo* classical resistance mutations can occur. The extent to which RR could oppose selection for the spread of classical resistance versus facilitate its emergence is unknown, yet this information could explain variation in the emergence and spread of resistance across populations. However, how the strength of selection and population size interact with plasticity to shape the evolution of any phenotype, is an unresolved area in evolutionary biology. Thus, the tractability of plasticity in parasite reproductive strategies and the step-changes to their environments (caused by drugs, vaccines, hosts shifts, etc.), could be harnessed to use malaria parasites as a model for general insight into how plasticity and evolution interact.

### 5.4 Evolution-proofing interventions

Evolutionary ecology can also inform how to make interventions more robust. For example, an intervention that aims to render one sex infertile intuitively seems sufficient to block transmission. Males seem the most tractable target given their greater vulnerability to drugs and oxidative damage [[48], [49], [50]]. However, if males are targeted, could parasites tap into their existing FI strategies to plastically ramp up male investment and partially compensate for the fitness loss caused by the intervention [48,63]? Although parasites exposed to the intervention will have lower fitness than untreated parasites, sex ratio adjustment (just like any resistance mechanism) will be selected for if it improves fitness compared to parasites that do not adjust sex ratio [48]. In contrast, parasites cannot evade an intervention that targets zygotes by adjusting sex ratio, for which the only option is elevating conversion rate, and this comes at cost to asexual replication. The constraint imposed by the need to protect asexual replication may make zygotes a more evolution-proof target [48]. Zygotes could be targeted directly or by targeting males and females in such a way that they can be mated but not develop into/beyond zygotes [48]. The latter approach has the advantage that even if some gametes escape unscathed, they are likely to mate with a damaged gamete which will render their offspring unviable [48].

### 5.5 Ecological traps

Once the cues that parasites use to detect a change in circumstance, and the molecular mechanisms involved in sensing are identified, it might be possible to develop interventions to trick parasites into making suboptimal sex ratio / conversion decisions (an “ecological trap”). If parasites base decisions on cues that are usually accurate indicators of their circumstances (Table 2; predictably plastic strategies), then mimics of cues can be administered to infections to coerce parasites into adopting the worst strategies for their actual circumstances. For instance, inducing malaria parasites to vastly increase conversion would reduce asexual population size, reducing the severity of symptoms and facilitating clearance of the infection. Ideally, parasites would be forced to convert to a single sex to avoid increasing transmission risk, but transmission could also be prevented (e.g. with bednets). It is possible that parasites have additional pathways for commitment to asexuals, males, and females, that prevent accidental terminal investment or all gametocytes differentiating into a single sex. Even if such safeguards could not be circumvented with an intervention, substantially reducing parasite fitness still has clinical and epidemiological benefit. Furthermore, targeting complicated environmental sensing mechanisms to manipulate parasite decision-making may be a relatively evolution-proof strategy. This is because selection may not allow parasites to ignore the misleading cues provided by the intervention due to the high costs associated with unresponsiveness to informative cues that feed into other phenotypes (Fig. 1).

## 6 Summary

Given that resistance to all front-line antimalarials has evolved, the need for new approaches is pressing and stimulating renewed interest in transmission-blocking interventions. The generally good fit between theoretical predictions for sex ratio and conversion rate – at least in model systems - gives optimism that such interventions can be made as evolution-proof as possible. However, evolutionary and mechanistic insight remain poorly integrated (Fig. 1). We contend that uncovering mechanisms for sensing state and environmental conditions are key to uniting these bodies of work. For example, identifying the cues parasites respond to will facilitate investigation of the molecular processes involved, and knowledge of mechanisms will facilitate elegant tests of fitness consequences. Tests of the basic predictions in natural infections of humans suggest that they largely apply to *P. falciparum*, which is not surprising given that the same principles shape reproductive strategies across the tree of life. Furthermore, opening the black box of reproductive strategies in other human parasites (such as the tractable *P. knowlesi)* is long overdue. Insight across species will offer opportunities to finesse theories for the specifics of their biology (such as long gametocyte maturation in *P. falciparum*), and future data sets collected with more sensitive, molecular tools offers unprecedented opportunities to test predictions.

## Author statement

Petra Schneider: Conceptualization, Writing - Original Draft, Writing - Review & Editing, Visualization.

Sarah E. Reece: Conceptualization, Writing - Original Draft, Writing - Review & Editing, Visualization, Funding acquisition.

## Funding sources

SER and PS are supported by the 10.13039/100010269Wellcome Trust [202769/Z/16/Z] and the 10.13039/501100000288Royal Society [UF110155; URF/R/180020].
